# Acceptability and Utility of a Digital Group Intervention to Prevent Perinatal Depression in Youths via Interactive Maternal Group for Information and Emotional Support (IMAGINE): Pilot Cohort Study

**DOI:** 10.2196/51066

**Published:** 2024-02-02

**Authors:** Keshet Ronen, Anupa Gewali, Kristin Dachelet, Erica White, Marimirca Jean-Baptiste, Yolanda N Evans, Jennifer A Unger, S Darius Tandon, Amritha Bhat

**Affiliations:** 1 Department of Global Health University of Washington Seattle, WA United States; 2 Division of Adolescent Medicine Seattle Children's Hospital Seattle, WA United States; 3 Department of Obstetrics and Gynecology Warren Alpert Medical School at Brown University Providence, RI United States; 4 Department of Medical Social Sciences Northwestern Feinberg School of Medicine Chicago, IL United States; 5 Center for Community Health Northwestern Feinberg School of Medicine Chicago, IL United States; 6 Department of Psychiatry and Behavioral Sciences University of Washington Seattle, WA United States

**Keywords:** perinatal depression, youth, mHealth, digital health, acceptability, utility, depression, pilot study, pregnancy, postpartum, prevention, cognitive behavioral therapy, psychoeducation, mixed methods, manage, mood, mobile phone

## Abstract

**Background:**

Perinatal depression (depression during pregnancy or the first year postpartum) affects 10%-25% of perinatal individuals, with a higher risk among youths aged <25 years. The Mothers and Babies Course (MB) is an evidence-based intervention for the prevention of perinatal depression, grounded in cognitive behavioral therapy, attachment theory, and psychoeducation.

**Objective:**

We developed a digital adaptation of MB (Interactive Maternal Group for Information and Emotional Support [IMAGINE]) and evaluated it in a pre-post mixed methods pilot among young perinatal people in the United States.

**Methods:**

IMAGINE was a structured digital group of up to 7 participants, with scheduled MB content and open discussion for 12 weeks, facilitated by a social worker. Scheduled content included asynchronous SMS text messages, graphics, prerecorded videos, mood polls, and optional weekly synchronous video calls. Eligible participants were pregnant or ≤80 days postpartum, aged 16 to 24 years, had access to a smartphone, spoke English, and had a Patient Health Questionnaire score <10. Participants were recruited throughout the United States from August 2020 to January 2021 through paid social media ads, in-person outreach at clinics, and respondent-driven sampling. Participants completed quantitative questionnaires at enrollment and 3 months, and qualitative interviews at 3 months. We determined uptake, acceptability (by Acceptability of Intervention Measure score), and utility (by use of cognitive behavioral therapy skills). We compared depression symptoms (by Patient Health Questionnaire score), social support (by abbreviated Social Support Behavior score), and perceived stress (by Perceived Stress Score) between enrollment and follow-up by paired 2-tailed *t* test.

**Results:**

Among 68 individuals who contacted this study, 22 were screened, 13 were eligible, and 10 enrolled, for an uptake of 76.9%. Furthermore, 4 (40%) participants were pregnant at enrollment. Participants had a median age of 17.9 (IQR 17.4-21.7) years, 6 (67%) identified as Black, 5 (56%) Latinx, and 6 (67%) using Medicaid health insurance. Further, 9 (90%) participants completed follow-up. Among these, the mean acceptability score was 4.3 out of 5 (SD 0.6) and all participants said they would recommend IMAGINE to a friend. Participants reported using a median of 7 of 11 skills (IQR 5-7 skills) at least half the days. We found no significant changes in depression symptoms, perceived stress, or social support. Qualitatively, participants reported one-to-one support from the facilitator, connection with other parents, and regular mood reflection were especially helpful aspects of the intervention. Additionally, participants reported that the intervention normalized their mental health challenges, improved their ability to manage their mood, and increased their openness to mental health care.

**Conclusions:**

This pilot study provides promising evidence of the acceptability and utility of IMAGINE among perinatal youths. Our study’s small sample size did not detect changes in clinical outcomes; our findings suggest IMAGINE warrants larger-scale evaluation.

## Introduction

Perinatal depression, defined as depression during pregnancy or up to 1 year after childbirth, affects an estimated 10%-25% of birthing people in the United States [1,2]. Untreated, perinatal depression can have long-term negative impacts on birthing parents and newborns, including elevated risk of suicide [3], preterm birth [4], low birth weight [5], and impaired infant attachment [6]. Young parents (aged 15 to 24 years), those with low income, and people of color experience elevated risks of perinatal depression [7,8].

Despite many programs aimed at reducing the incidence of adolescent pregnancy in the United States, nearly 23% of all pregnancies occurred in people aged 15 to 24 years in 2020 [9]. Young parents are more likely to have risk factors that place them at greater risk for perinatal depression, including unplanned pregnancy, social isolation, and intersecting social determinants of health [10,11]. Many barriers to mental health care exist for perinatal youths, including lack of financial resources to pay for treatment, lack of access to or money for transportation, and difficulty finding childcare and missing work to attend appointments [8]. Young parents are therefore a key group needing innovative and tailored perinatal mental health support.

Evidence-based interventions have been developed to prevent and treat perinatal depression. The Mothers and Babies Course (MB) is a cognitive behavioral therapy (CBT) program developed for low-income racial minority women in the United States [12]. MB can be delivered to individuals or groups and is focused on building participants’ skills in modifying their thoughts, social contact, and pleasant activities. Further, 4 randomized controlled trials of the MB program found it reduced depressive symptoms in the perinatal period, and it has been rolled out at scale in the United States through home visiting programs [13-18]. MB has been recognized by the US Preventive Services Task Force as an evidence-based intervention that should be recommended for individuals at high risk of perinatal depression [19]. Despite these advances, there are barriers to accessing interventions such as MB, due to regional provider shortages, lack of transportation and childcare, clients’ difficulty committing to a prespecified time to attend sessions, and experienced or internalized stigma of seeking mental health care [20]. These barriers may be higher among those at the highest risk of perinatal depression, and barriers were further exacerbated by the COVID-19 pandemic and consequent physical distancing protocols [21-24].

Mobile phones can be used to offer more accessible mental health support to young parents (known as mobile health [mHealth]). mHealth interventions have shown promising results in improving perinatal mental health [25-30], and young people in the United States are an ideal audience for such programs: nearly all Americans aged 18 to 29 years own a phone, and 96% own a smartphone [31]. mHealth can eliminate the need to travel to appointments, and asynchronous mHealth programs allow patients to access care within their schedules, which can be especially helpful to parents [32]. Group mHealth interventions allow new parents to connect with and share ideas with peers, which can lower feelings of social isolation and increase social support and mental well-being [33].

We developed a digital adaptation of MB for the prevention of perinatal depression in youths, named Interactive Maternal Group for Information and Emotional Support (IMAGINE) [34]. In this paper, we present a mixed methods evaluation of a pilot study of the IMAGINE intervention, assessing the uptake, acceptability, and utility of IMAGINE.

## Methods

### Study Design

We conducted a single-arm pilot study with pre-post mixed methods evaluation.

### Study Population and Recruitment

Participants were eligible to participate in the IMAGINE pilot if they were: pregnant or ≤180 days postpartum, aged 16 to 24 years during pregnancy, had daily access to a smartphone, and were comfortable conducting study visits and reading and responding to social media messages in English. Individuals who exhibited elevated depression symptoms at screening (Patient Health Questionnaire [PHQ-9] score ≥10 [35]) were excluded and referred to individual clinical care.

Participants were recruited between August 2020 and January 2021, through 3 main methods. First, study information was shared through paid, targeted advertisements on Instagram and Facebook throughout the United States. Specific parameters used to target participants were: female sex, aged 16 to 25 years, located anywhere in the United States. Second, this study’s team identified health care providers and community-based organizations in several cities in the United States (Seattle, WA; Olympia, WA; Philadelphia, PA; and Temple, TX) and provided materials for staff at these organizations to promote this study by distributing flyers. Finally, we used respondent-driven sampling [36] to encourage participants who enrolled in this study to invite their peers to participate; a financial incentive of US $20 was offered to the referring participant if their referred peer enrolled.

Potential participants who learned about this study through any method contacted this study by phone call, text message, email, Instagram message, or by sending a message through this study’s website. Study staff then contacted potential participants to conduct eligibility screening by phone or video call. If participants were ineligible due to elevated depression symptoms at screening, study staff shared the National Crisis Line phone number and offered support to find treatment near their location. This study’s team psychiatrist was available to support linkage with resources.

### Informed Consent

Informed consent was obtained by study staff for screening and enrollment. Participants provided verbal consent to participate in eligibility screening. Eligible participants provided written consent for study enrollment, using a web-based consent form on REDCap (Research Electronic Data Capture; Vanderbilt University) [37].

### Study Visit Schedule

Participants enrolling in the IMAGINE pilot attended 2 study visits by video call: enrollment and 3-month follow-up after completion of the intervention. Quantitative data were collected at enrollment and follow-up using a REDCap web-based questionnaire. Qualitative interviews were additionally conducted at follow-up.

### Intervention

The IMAGINE intervention was a facilitated digital group adaptation of the evidence-based MB program [12], developed through a human-centered design process described elsewhere [34]. MB content focuses on engagement in pleasant activities, healthy thinking, and social support, and is grounded in CBT, attachment theory, and psychoeducation. IMAGINE was delivered using the messaging platform, Slack (Slack Technologies, Salesforce Inc), to groups of up to 10 participants, facilitated by a member of this study’s team with Master’s-level training as a social worker. Groups ran for approximately 12 weeks. Participants were grouped based on the timing of enrollment: groups were filled sequentially as participants enrolled. Guided by our formative work [34], the intervention consisted of multimedia adaptation of MB content, delivered through 5 components. First, MB session content was delivered asynchronously, through short SMS text messages, summary graphics, and prerecorded videos sent approximately 4 times per week. Messages were designed to promote group discussion or personal reflection. Participants were encouraged, but not required, to participate in group discussions by sending messages and reacting to other participants’ messages, at a time that was convenient for them. Second, an automated “mood poll” was sent to each participant individually 3 times per week, prompting the participant to reflect on their mood, activities, thoughts, and social contacts. Third, the facilitator was available for individual messaging through Slack. This was used for the facilitator to answer questions and send messages when a participant showed low engagement in other parts of the intervention. Fourth, participants could send messages on topics beyond the MB curriculum, through separate Slack “channels” (parallel conversations all members had access to): “ask an expert,” where participants could send questions for members of this study’s team with expertise in obstetrics and psychiatry; “random,” where participants could share any content; and “references,” where the facilitator posted graphics summarizing intervention content and links to resources. Fifth, in addition to asynchronous messaging content, the facilitator held a weekly 1-hour synchronous group video call, using the videoconferencing platform, Zoom (Zoom Video Communications, Qumu Corporation). Participation in the call was optional, to reduce barriers to participation due to scheduling and attendance challenges. No new content was delivered on the call, but participants could ask questions, share experiences, and receive support from the facilitator and other group members. Intervention content was developed in advance of the intervention and manually sent by the facilitator, except for mood polls, which were automatically scheduled within Slack. The facilitator could exercise discretion in message pacing based on participant feedback and questions during the intervention period. All 5 elements of the intervention were considered to be active components in engaging MB’s mechanism of action.

### Quantitative Data Collection

#### Recruitment Log

A spreadsheet was used by study staff to record participants who contacted the IMAGINE study and their completion of eligibility screening.

#### Screening Questionnaire

Screening was completed verbally by phone or video call and responses were entered by study staff into an electronic questionnaire using REDCap, hosted at the University of Washington [37]. The screening questionnaire ascertained pregnancy status, age, access to a smartphone, comfort in English, and depression symptoms by PHQ9.

#### Enrollment Questionnaire

Enrollment of eligible participants was conducted either immediately following consent or at a separate scheduled visit, based on participant preference. The enrollment questionnaire was administered using REDCap by study staff through Zoom or phone calls. If conducted by video call, study staff screen-shared the REDCap questionnaire and read each question aloud so the participant could see and hear the questions and responses; data were entered by study staff. The enrollment questionnaire ascertained demographic characteristics and technology access. It also included an abbreviated 12-item version of the Social Support Behavior (SSB) instrument [38] to ascertain social support (score range 5-60). We used an abbreviated version of SSB to reduce the burden of data collection on participants. This version of the SSB has not been psychometrically validated. We used the Perceived Stress Score (PSS-4) instrument [39] to ascertain perceived stress (score range of 0-16). This instrument has previously been used in MB studies and is recommended for comparability [40,41]. Instrument reliability was previously reported as Cronbach α .72 [39].

#### Follow-Up Questionnaire

A follow-up visit was conducted at 3 months. The week after the completion of the IMAGINE intervention, a member of this study’s team other than the group facilitator contacted participants and arranged a follow-up study visit, conducted via Zoom video conference. The follow-up electronic questionnaire was administered using REDCap and assessed pregnancy status, PHQ9, abbreviated 12-item SSB, PSS-4, and acceptability of IMAGINE via the Acceptability of Intervention Measure (AIM) [42]. The AIM scale is 4 Likert scale questions with possible responses scored 1-5: completely disagree, disagree, neither agree nor disagree, agree, and strongly agree. The score is calculated as the mean numerical value of the responses across the 4 items. Instrument internal consistency and validity were previously reported as Cronbach α .85 and confirmatory factor analysis loadings 0.75-0.89 [42]. Participants were also asked how often they had used key CBT skills over the past month, using a Likert scale with possible responses: not at all, a few times, half the days, most of the days, and every day. The following skills were asked about mood tracking, engaging in pleasant activities, overcoming obstacles to engage in pleasant activities, thought interruption to reduce harmful thoughts, designated worry time to reduce harmful thoughts, time projection to imagine a better time in the future, self-instruction, positive contact with others, soliciting positive support from others, and using assertive communication. These questions were modeled on the core CBT components of the MB program [17]. If a participant reported the use of a skill, they were asked how helpful the skill was, using a Likert scale with possible responses: not helpful at all, somewhat helpful, or very helpful. Study staff screen-shared the REDCap questionnaire, as in the enrollment visit.

#### Engagement data

We quantitatively assessed engagement in the different components of IMAGINE. Messaging data from all Slack channels was exported as an HTML file. Message counts for each group member were determined by searching for each member’s username and counting the number of occurrences in the file. Completion of automated mood polls was determined based on reports from the Polly tool within Slack that was used to send polls. Attendance of each group member in Zoom calls was recorded by the facilitator.

### Quantitative Data Analysis

We calculated descriptive measures of uptake, acceptability, and utility. Uptake was defined as the percentage of screened, eligible participants who enrolled in the intervention. Acceptability was determined based on self-report responses to the AIM. Utility was defined as the percentage of participants who reported using each CBT skill discussed in MB at least half the time. Additionally, among those who used each skill at least half the time, the percentage of participants who found it to be helpful was calculated.

We determined pre-post change in indicators of mental wellness. Depression symptoms were determined by PHQ9 score, calculated according to instrument guidelines, with a possible range of 0-27. Social support was calculated using the abbreviated SSB, as the sum score over all questions referring to family support and separately those referring to friend support. The mean and SD for each score were summarized for each time point. Scores at the 2 time-points were compared by paired 2-tailed *t* test. RStudio (version 2023.06.0+421; Posit) was used for all data analysis.

### Qualitative Data Collection

Qualitative In-Depth Interviews (IDIs) were conducted with all study participants at the 3-month follow-up visit. IDIs were conducted virtually over Zoom using a semistructured interview guide designed by this study’s team. The guide explored participants’ experience in the intervention, utility and potential improvements to each component of the intervention, level of engagement with intervention content, barriers to participation, and recommended improvements for future intervention implementations.

### Qualitative Data Analysis

We conducted a thematic analysis of IDIs using a mixture of inductive coding driven by themes emerging from the transcripts and deductive coding based on themes from the interview guide. Qualitative analysis focused on the perceived acceptability and mental health impact of the IMAGINE intervention as well as recommendations for future iterations. First, 3 members of this study’s team (KR, EW, and AG) read all transcripts and separately developed initial codebooks based on themes that emerged from the transcripts and were explored in the interview guide. Initial codebooks were compared and discussed to create an agreed-upon combined codebook. Transcripts were then coded by 2 analysts (EW and AG) using Dedoose (Sociocultural Research Consultants, LLC) software [43]. Disagreements between analysts were resolved through discussion.

### Ethical Considerations

The IMAGINE study was approved by the University of Washington Institutional Review Board (STUDY00008278). All participants provided informed consent for eligibility screening, exposure to the intervention, and data collection. Waivers were obtained for written documentation of informed consent and parental consent for adolescents younger than 18 years.

## Results

### Participant Flow and Intervention Uptake

**Figure 1:**
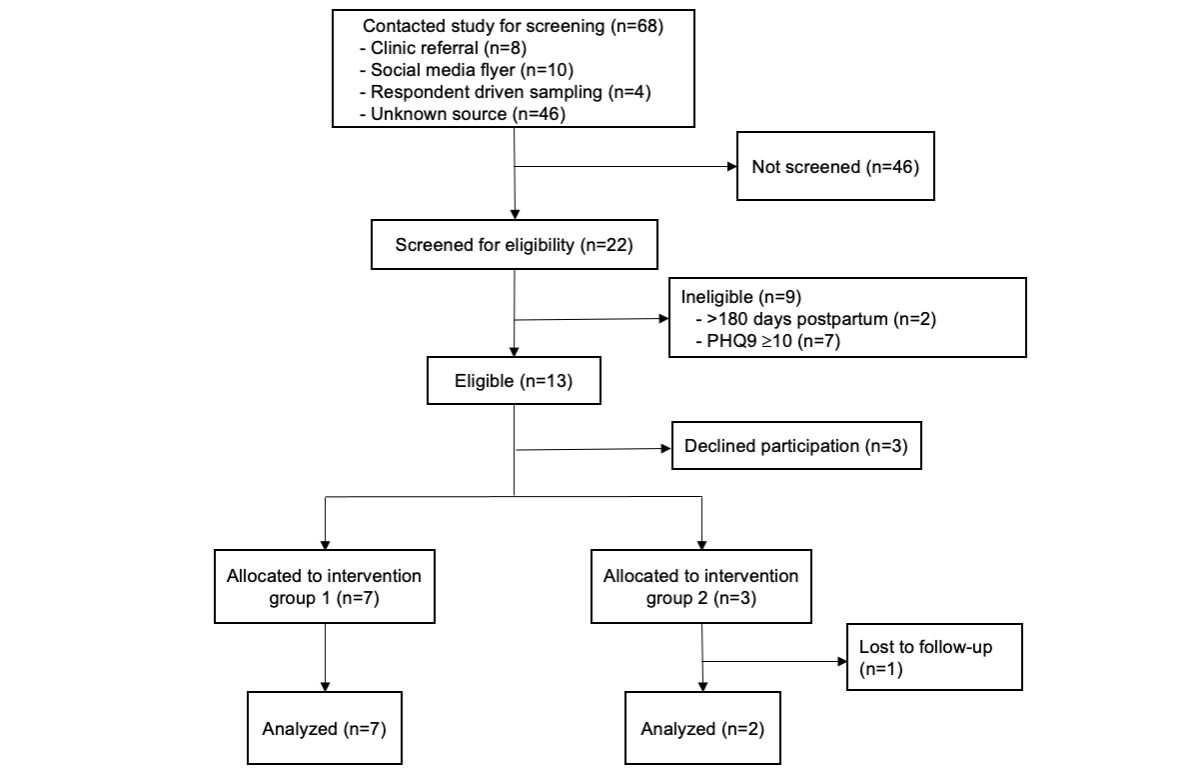
Participant flow. PHQ-9: Patient Health Questionnaire.

Figure 1 summarizes participant flow from contacting this study to completing screening, enrolling in this study, and completing follow-up. In total, 68 individuals contacted this study between October 16, 2020, and January 29, 2021. Of these, 22 were assessed for eligibility, while 46 individuals did not complete screening due to challenges scheduling screening calls or participants declining to complete screening. Of the 22 who were assessed, 13 were eligible and 9 were ineligible, most (n=7, 77.8%) due to elevated depression scores warranted referral to individual care. In total, 3 eligible participants declined participation and 10 eligible participants were enrolled in the pilot, for an uptake of 76.9% (10/13 eligible participants). Participants were divided into 2 intervention groups: 7 in group 1 (active December 2020 to February 2021) and 3 in group 2 (active February to May 2021). While groups could be up to 10 participants, we elected to initiate the first group when 7 participants had been enrolled to minimize participants’ wait, while recruitment of the remaining participants continued. Of the 10 study participants, 9 completed the 3-month follow-up questionnaire and IDI.

### Participant Demographic Characteristics

Demographic characteristics of enrolled participants are summarized in Table 1. All participants identified as female, and the median age was 17.9 (IQR 17.4-21.7) years. Of 9 participants who provided their race or ethnicity, 6 (67%) identified as Black, 5 (56%) identified as Latinx, 1 identified as an unlisted category, and 2 (22%) identified as White. Further, 4 (40%) participants were bilingual and 5 (50%) had completed at least a high school diploma or general education development. In total, 4 (40%) participants were pregnant at the time of this study and 6 (67%) used Medicaid health insurance. All but one of the participants reported that they were stably housed.

**Table 1:** Participant baseline characteristics.

Participant characteristic		Participants	
Age (y; N=10), median (IQR)		17.9 (17.4-21.7)	
**Sex** **(N=10), n (%)**			
	Female	10 (100)	
**Race or ethnicity^a^ (n=9), n (%)**			
	Black	6 (66.7)	
	Latinx	5 (55.6)	
	Not listed	1 (11.1)	
	White	2 (22.2)	
**Pregnancy status** **(N=10), n (%)**			
	Pregnant at the time of enrollment	4 (40)	
**English proficiency** **(N=10), n (%)**			
	Fluent	10 (100)	
**Bilingual** **(N=10), n (%)**			
	Yes	4 (40)	
**Education level** **(N=10), n (%)**			
	9-12th grade	5 (50)	
	High school diploma or GED^b^	1 (10)	
	>High school	4 (40)	
**Employment** **(N=10), n (%)**			
	No	8 (80)	
	Part-time (<40h/wk)	1 (10)	
	Full-time (40h/wk)	1 (10)	
**Health insurance status** **(N=10), n (%)**			
	Medicaid	7 (70)	
	Employer-provided private	3 (30)	
**Housing status** **(N=10), n (%)**			
	Stably	9 (90)	
	Unstably	1 (10)	

^a^Race or ethnicity categories are not mutually exclusive.

^b^GED: general education development.

### Intervention Acceptability and Utility

#### Quantitative Assessment

Among the 9 participants who completed follow-up, we found a mean acceptability score of 4.3 out of 5 (SD 0.6) on the AIM questionnaire. All participants reported that they would recommend IMAGINE to a friend. When asked about the use of core CBT skills covered in MB, all participants reported engaging in “playing with baby,” “contact with others,” and “talking to/contacting someone who has been a positive support for self and baby” skills during at least half the days in the prior month (Table 2). The majority reported using the following skills at least half the days: “mood tracking” (n=6, 66.7%), “engaging in pleasant activities” (n=6, 66.7%), “overcoming obstacles to doing pleasant activities” (n=6, 66.7%), “thought interruption to reduce harmful thoughts” (n=5, 55.6%), “using time projection to imagine a better time in the future” (n=6, 66.7%), and “using self-instruction to give oneself helpful directions” (n=7, 77.8%). When asked about the helpfulness of each skill, 100% of participants who used them (n=9) reported they were helpful (Table 2). Of the 11 skills we asked about, participants reported using a median of 7 (IQR 5-7) skills at least half the days.

**Figure 2:**
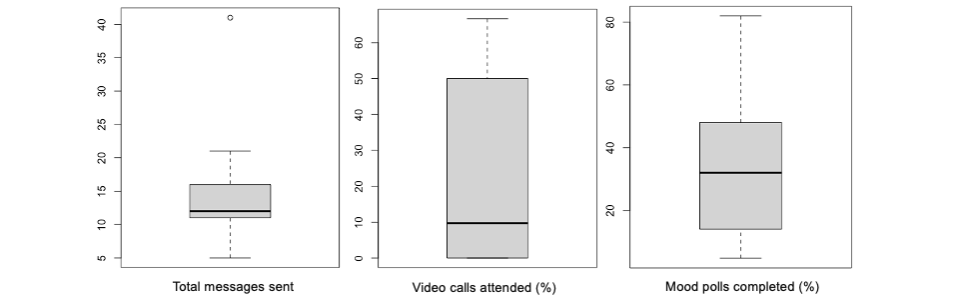
Participant engagement in the intervention.

We also analyzed participant engagement in the intervention. Figure 2 summarizes 3 measures of engagement: the number of SMS text messages sent on all Slack channels over the course of the intervention, the proportion of video calls attended, and the proportion of mood polls completed. Participants sent a median of 12 (IQR 11-15.8) messages during the 12-week intervention period, attended a median of 9.7% (IQR 0%-45.8%) of the weekly video calls, and responded to a median of 32% (IQR 14.5%-47%) of the mood polls. Levels of engagement, particularly messaging, generally decreased over time but increased at intervention close (Multimedia Appendix 1).

**Table 2:** Frequency and helpfulness of MB^a^ skill use.

Skill	Participants who used skill for half of the time or more (n=9), n (%)	Participants who found skill helpful^b^, n (%)
Kept track of mood	6 (66.7)	6 (100)
Engaged in pleasant activities	6 (66.7)	6 (100)
Overcame obstacles to engage in pleasant activities	6 (66.7)	6 (100)
Used thought interruption to reduce harmful thoughts	5 (55.6)	5 (100)
Used worry time to reduce harmful thoughts	1 (11.1)	1 (100)
Used time projection to imagine a better time in the future	6 (66.7)	6 (100)
Use self-instruction to give yourself helpful directions	7 (77.8)	7 (100)
Played with baby	9 (100)	9 (100)
Had positive contact with others	9 (100)	9 (100)
Talked to or contacted someone who has been a positive support to yourself or baby	9 (100)	4 (100)
Made a request using assertive communication	3 (33.3)	3 (100)
Met a new person who can provide support for you and your baby^c^	4 (44.4)	4 (100)

^a^MB: Mothers and Babies Course.

^b^Among those who reported using the skill; includes “somewhat helpful” and “very helpful” responses.

^c^Responses to this question asked for the number of new people who met who can provide support. This n represents the number of those who met at least 1 new support person.

#### Qualitative Assessment

IDIs explored the acceptability and perceived utility of IMAGINE. Participants highlighted several aspects of the intervention that were especially well-received (Textbox 1). Support from both the facilitator and other participants was viewed as beneficial. Most participants (n=8) reported valuing the connection with the facilitator and feeling that they could go to them for guidance and support. Participants (n=7) highlighted that support from other young parents in the program normalized and validated their experiences; several participants spoke about the value of connecting with others in similar situations to them. Mood polls were mentioned by all 9 participants as an impactful component of the intervention, with participants valuing the opportunity to reflect on their emotional state. In total, 2 participants highlighted the value of the intervention’s flexibility, both that the asynchronous design allowed them to access content at a time and pace that worked best for them, and that the facilitator made adaptations to respond to participant needs during intervention delivery.

**In-Depth Interview themes related to intervention acceptability.**

**Support from the study facilitator**

- When I had something come up or just anything, like, I would just talk to her, or if I had a question, I would talk to her. I asked her the question, and she would like answer the best way that she could or stuff. And I was just like, that's really helpful.
  [Participant 5, aged 21 y, postpartum]
- I had a kind of Facetime with her one time, just us, because the other girls weren’t able to make it. But that was very - I really appreciated that. It was kind of something that I didn't know that I needed, but she willing to like, hear me out and ask the right questions for me. So I think that was pretty awesome of her.
  [Participant 7, aged 23 y, pregnant]

**Connection with other participants**

- Seeing other women, not necessarily the facilitator, but being around the women that are going through the same things that you're going through, like that are also pregnant and just had babies, that makes a difference. That allows the connections that you form are a lot stronger and a lot tighter
  [Participant 9, aged 23 y, postpartum]
- We all went through this together and I felt like we all kind of grew together and, you know, they could, we could all relate to each other. And I didn't really have many mom friends I guess before this.
  [Participant 17, aged 17 y, pregnant]

**Mood polls**

- Normally you don't really reflect on your day unless you have a bad day. So I just like the way how it got me thinking, ‘Oh, well, I did have a good day but I didn't notice it because it wasn't a bad day’ if that makes sense.
  [Participant 12, aged 23 y, postpartum]
- I think they were helpful just because it was like a second to self-reflect, maybe look in the real world. Sometimes they ask you how you doing but it's kind of difficult to explain to someone, but when you're doing it for yourself, I think it can be a little bit more honest.
  [Participant 7, aged 23 y, pregnant]

**Intervention flexibility**

- At the beginning I think it started off slow and then she was sending a lot of messages, every day, so I wouldn't get time to read them. I’d have to go back a lot, and then that's something that I told her, and so she slowed down on the number of messages, so she sent them every other day, instead of every single day.
  [Participant 17, aged 17 y, pregnant]
- Having lots of interactions in written form gave everyone an opportunity to share their perspective and their experiences and what works for them, and we didn't all have to be right then and there, like I could look at it at two o'clock in the morning and another girl could look at it at two o'clock in the afternoon.
  [Participant 9, aged 23 y, postpartum]

### Mental Wellness

#### Quantitative Assessment

Summary statistics for mental wellness outcomes are presented in Table 3 and individual participant trajectories are displayed in Multimedia Appendix 2. We compared depression symptoms (by PHQ-9), perceived stress (by PSS-4), and social support (by abbreviated SSB) between enrollment and follow-up. No significant changes in scores were found. At enrollment, the median PHQ-9 score was 4.0 out of 27 (IQR 2.0-5.0), compared with 2.0 (IQR 2.0-3.0) at follow-up (*P*=.25); 6 of 9 participants demonstrated reductions in PHQ-9 scores from baseline to follow-up. Median PSS-4 at enrollment was 8.0 out of 16 (IQR 8.0-10.0) and 9.0 out of 16 (IQR 8.0-10.0) at follow-up (*P*=.46). The median social support score related to participants’ family was 50.0 out of 100 (IQR 42.0-53.0) at enrollment, and 48.5 out of 100 (IQR 47.5-50.5) at follow-up (*P*=.11). The median social support score for friends was 51.0 out of 100 (IQR 47.0-54.0) at enrollment and 50.0 out of 100 (IQR 46.0-53.0) at follow up (*P*=.88).

**Table 3:** Change in participant mental wellness from baseline to follow-up.

Measure	Enrollment, median (IQR)	Follow-up, median (IQR)	*P* value
Depression (PHQ-9^a^)	4.0 (2.0-5.0)	2.0 (2.0-3.0)	.25
Perceived Stress Score (PSS-4)	8.0 (8.0-10.0)	9.0 (8.0-10.0)	.46
Social support	Family: 50.0 (42.0-53.0); friends: 51.0 (47.0-54.0)	Family: 48.5 (47.5-50.5); friends: 50.0 (46.0-53.0)	Family: .11; friends: .88

^a^PHQ-9: Patient Health Questionnaire.

#### Qualitative Assessment

In IDIs, participants highlighted 3 ways they perceived participating in IMAGINE benefited their mental health (Textbox 2). First, participants (n=5) expressed that being in a group with other young parents normalized and validated their experiences, which helped them feel more connected with others and strengthened their belief that they could make changes to improve their mood. Second, several participants (n=5) noted that they had gained skills in regulating their emotions. Some stated that they still used some of the mood monitoring and management techniques discussed in the program after it had ended. Participants highlighted that they had few opportunities in their day-to-day lives to reflect on their mood and that there was value and ease in reflecting “for yourself” through the digital platform, rather than to another person who may not understand their feelings. Participants also commented that they learned to pay closer attention to emotions in the middle of the spectrum (ie, not crisis or elation), and that helped them monitor when and why their mood changed. Third, several participants (n=6) stated that they would be more open to mental health services in the future after they completed IMAGINE. Some common feedback from participants related to this was that it helped them realize they needed to return to psychotherapy if they had sought it in the past, or for those without prior experience, IMAGINE helped normalize some of the unknown or off-putting aspects of mental health care. It became more familiar to discuss moods and feelings with others, which increased their openness to seeking care.

**In-Depth Interview themes related to mental health impact.**

**Normalized experiences**

- It helped me, like, open up a little more, and realize that I’m not the only person suffering, and that there's more people like me that I can talk about it with. I don't have to, you know, suffer by myself.
  [Participant 17, aged 17 y, pregnant]
- [IMAGINE helped me] understand… my emotions right now were normal… Before, I felt like I was broken or I needed to be fixed or there's something wrong with me and now… there's nothing wrong with me. [I’m] normal.
  [Participant 9, aged 23 y, postpartum]

**Gained emotional regulation tools**

- I had a pregnancy before that and it was also a preterm birth. And so, that baby passed away. And so it was really hard for me. I was really depressed, and so I know that if I would have had like the Imagine group it like it would help it would have helped me to manage my, my emotions and stuff.
  [Participant 5, aged 21 y, postpartum]
- I definitely added a handful more tools to my arsenal to be able to calm myself down, stay calm, or not become overwhelmed with everything that's going on… I've been using these - to me they're like lower tier ways of coping. So it's like when I'm not at a ten, I'm at like a five, I can use these.
  [Participant 9, aged 23 y, postpartum]
- Reading the mood tree1.. identifying the stressors… how to regulate it, different forms of communication, just all these different things for your mood and focusing on that. It just helped me to realize taking myself is a priority. And not just my baby and making sure she’s okay but making sure I'm okay as well.
  [Participant 17, aged 17 y, pregnant]

**Opened to mental health services in the future**

- I like to bottle up my anger or my problems and they explode on the wrong person at the wrong time so that being in a group kind of made me realize like I need to go back to counseling. I was already in counseling but I don't feel like I was taking it as serious but when I sat back and realized… No, I do need to get it together, that's when I was like, I'mma just go back to counseling.
  [Participant 14, aged 18 y, pregnant]
- When you talk to [my family] about… feeling depressed and you're not mentally okay they’re, like, “oh that's not real, it's fake, it's all in your head”... And so, [IMAGINE] just helped me to think that there is help, and it is real, and that it's not just in your head.
  [Participant 5, aged 20 y, postpartum]

### Recommended Improvements

IDIs explored participants’ barriers to intervention participation and recommended improvements to IMAGINE. Frequently identified barriers to participation included being too busy, introversion, and low engagement from other members (Textbox 3). Participants (n=7) mentioned being unable to read all the messages or join Zoom meetings because they were working, in school, or busy with parenting responsibilities. Introversion was mentioned by a few participants (n=3), and 1 participant stated that a reason she did not participate in more Zoom calls was the thought that she was expected to be on camera, which she found uncomfortable. Limited engagement from other group members was discussed as a deterrent to engagement: participants (n=6) felt they would have engaged more and benefited more if synchronous and asynchronous conversations had included more voices. In total, 2 participants also reported technology challenges, both of which were resolved.

Participants shared recommendations to address their barriers to engagement (Textbox 3). Several participants (n=8) recommended changes to the frequency and timing of the synchronous Zoom calls to facilitate their attendance. Calls were scheduled each week based on polls with the group to identify the optimal time. Participants suggested that offering calls in the evenings, during the weekends, or announcing the schedule for all calls at the start of the program rather than scheduling them week-by-week could improve attendance. A few participants suggested offering multiple call times each week. Some participants (n=3) also requested more onboarding at the start of the group, including more orientation to the Slack platform and more icebreakers and rapport-building activities with other group members to encourage greater comfort and collective engagement in the group. While IMAGINE was designed to be virtual and COVID-19 restrictions meant in-person contact was not possible at the time, a common piece of feedback (n=7 participants) was to have at least one in-person meeting or offer a hybrid version of IMAGINE to deepen relationship-building with other group members.

**In-Depth Interview themes related to barriers to participation and recommended improvements.**

**Barriers**

- Being busy
  I couldn't read or join in on the calls, because I had started back working. [Participant 24, aged 16 y, pregnant]
  I started working like right after I got into [the study], and I didn't know when I was actually going to be available… Some of the polls or some of the Zooms I wasn't even able to join, because I work. [Participant 18, aged 19 y, pregnant]
- Introversion
  I’m a shy person. So that’s what, I just want to push myself more to be able to, you know, do more things like this because this is like out of my comfort zone. [Participant 17, aged 17 y, pregnant]
  Being on video is just not for me, I’m just not gonna keep with this. [Participant 12, aged 23 y, postpartum]
- Low engagement from other study participants
  It would have been better if everybody, you know, responded in and reacted. But it was a good thing to still be able to have somebody there talking to us, even though we weren’t always responding. [Participant 20, aged 18 y, postpartum]
  I just wish more people participated, because even in the zoom calls like sometimes I would be the only person that would join, or it would just be one or two other moms. [Participant 17, aged 17 y, pregnant]
- Issues with technology
  My phone like we started and it kind of deleted the app so I was just like, whoa. Okay. [Participant 5, aged 21 y, postpartum]
  I had to switch [cell phones], so I had turned in a phone that I was leasing for Sprint and then I had an old phone, but I couldn’t do certain things on it. Like I couldn’t download a lot of apps because I didn’t have the latest iOS and then, when I got the new phone, I had to wait to switch carriers. It was a lot going on, when I was trying to get a new phone. [Participant 12, aged 23 y, postpartum]

**Recommended improvements**

- More flexibility for Zoom calls
  I didn’t really get to join any of the zoom meetings because I was usually busy… I wish I could have. [Participant 5, aged 21 y, postpartum]
  If we had more zoom calls like twice a week, that would be really great for me. [Participant 7, aged 23 y, pregnant]
- Hybrid or in-person meetings
  I think that if we have more in person, it would have been better because it's more something that you can engage in more than just being online. [Participant 20, aged 18 y, postpartum]
  I feel if you were to go in person and meet everyone, I think that'd be great. You get to interact. [Participant 5, aged 21 y, postpartum]
- More onboarding
  At the very end, it was really nice because we were kind of more comfortable with each other, but at the beginning it was, understandably, a little bit awkward. We don’t know each other, but I think we could have benefited with some more icebreakers or some more like getting to know each other, rather than just kind of jumping in and expecting [us] to be open to each other. Because not everybody is willing to, you know, put themselves in that situation where they kind of have to be vulnerable. [Participant 7, aged 23 y, pregnant]
  Me personally I didn't like the Slack platform just because I'm not really tech savvy I guess you could say, and it was a lot of different components to the app, so it's kinda like I'm still trying to learn how to use it. [Participant 14, aged 18 y, postpartum]
- Low engagement from other study participants
  It would have been better if everybody, you know, responded and reacted. But it was a good thing to still be able to have somebody there talking to us, even though we weren’t always responding. [Participant 20, aged 18 y, postpartum]
  I just wish more people participated, because even in the zoom calls like sometimes I would be the only person that would join, or it would just be one or two other moms. [Participant 17, aged 17 y, pregnant]

## Discussion

### Principal Results

In this mixed methods pilot study, we found that IMAGINE, a novel digital adaptation of the evidence-based MB course, had high acceptability and perceived utility. Uptake of IMAGINE, defined as the proportion of eligible participants who enrolled, was high. However, many individuals who initially contacted this study did not complete the screening process, suggesting these screening procedures presented barriers. These barriers may include the need to arrange a call with study staff to complete screening; it is possible the reach would be higher in a lower-barrier delivery model where clients could self-register in the group without arranging a screening call. We also found that a substantial proportion of screened participants were ineligible, most commonly due to elevated PHQ-9 scores. While our study’s focus was on the prevention of perinatal depression, this observation suggests that IMAGINE may also be appealing to individuals who are already experiencing elevated symptoms of depression; this observation is important in considering potential future applications of IMAGINE.

Acceptability scores were high and all participants stated they would recommend IMAGINE to a friend. Quantitative measures of engagement in the intervention, such as frequency of messaging and attendance of video calls, demonstrated low-moderate engagement. Nevertheless, most CBT approaches discussed in the IMAGINE intervention were used frequently and reported to be useful by the majority of participants. Qualitatively, participants reported one-to-one support from the facilitator, connection with other parents, and regular opportunities to reflect on their mood through mood polls were especially helpful aspects of the intervention. Additionally, participants reported that the intervention normalized their mental health challenges, improved their ability to manage their mood, and increased their openness to mental health care. We found no significant changes in depression scores, perceived stress, or perceived social support, although our study’s small sample size was not powered to detect changes in these outcomes.

Participants also made recommendations for improvements to the IMAGINE intervention in future iterations, including more opportunities for synchronous connection with other group members, additional rapport-building activities, and further simplification of message content.

### Comparison With Prior Work

Our findings add to a small but growing body of literature on the use of digital interventions to prevent perinatal depression. While several studies have found that internet-delivered CBT is effective in the treatment of depression, including in the perinatal period [28,44-49], digital interventions for the *prevention* of perinatal depression have been less well studied [30,50].

Our findings are consistent with data from in-person MB. The levels of CBT skill use that IMAGINE participants reported were similar to that reported in a cluster randomized trial of in-person MB [51]: 66% (n=6) of IMAGINE participants reported that they engaged in pleasant activities and 100% (N=9) talked to or contacted someone who has been a positive support, compared with 78% and 80% respectively in Tandon et al’s [51] study previously adapted MB to a self-guided web-based format with informational pages, audio and video clips, and worksheets that follow MB modules in English and Spanish. A pilot randomized trial of the intervention, named Mothers and Babies Online Course, found nonsignificant improvement in depression symptoms; this study’s power was limited by sample size and the authors commented that facilitator guidance may improve uptake and efficacy [25,52]. The IMAGINE intervention differs from the Mothers and Babies Online Course in its inclusion of a facilitator and group delivery format, both of which were viewed as helpful components by participants in our pilot. Our findings of high acceptability, low-moderate engagement in intervention content, and high perceived utility are consistent with those of Barrera et al [25], suggesting digital delivery of MB with and without facilitation is a promising strategy that warrants further evaluation.

### Strengths and Limitations

Our study has several strengths. The IMAGINE intervention was developed through systematic adaptation of the evidence-based MB course that prioritized fidelity to core MB components while adapting to participants’ design recommendations [34]. Our recruitment strategy feasibly reached potential participants across the country. Our evaluation is strengthened by including measures of CBT skill use that align with the mechanism of MB and were used in previous MB studies, allowing comparison of our findings with other MB studies. Furthermore, our study explored the experiences of young perinatal people, whose often marginalized perspectives are critical to developing responsive interventions. Guided by the principles of human-centered design [53], we collected in-depth insights from users before and after our pilot to drive future iteration and improvement of the intervention.

The primary limitations of our study are its small sample size and nonrandomized design. This study was not powered to evaluate the intervention’s effect on mental health outcomes, and pre-post comparisons are susceptible to confounding by changes over time that are not attributable to the intervention. Additionally, recruitment was primarily through Facebook and Instagram, which were selected for participants who were already using social media platforms and may be more open to a digital intervention. Due to resource constraints for this pilot study, our eligibility criteria included the ability to read and write in English, which systematically excluded non-English speakers, whose needs may differ. Future evaluations should address the limitations of this study by achieving a larger sample size, employing a randomized design, recruiting from nonsocial media sources, and developing translations in additional languages, particularly Spanish, in which MB materials already exist.

### Conclusions

This pilot study provides promising evidence of the acceptability and utility of a digital group adaptation of MB among perinatal youths. These findings support further development and evaluation of the IMAGINE intervention to increase access to evidence-based interventions for the prevention of perinatal depression. Future iterations of IMAGINE will incorporate user recommendations from this study and use randomized powered evaluations to test clinical impact.

## Appendix

Multimedia Appendix 1 Participant interaction over time (group 1 and 2 combined).

Multimedia Appendix 2 Participant trajectories of mental wellness outcomes.

## Abbreviations

AIM: Acceptability of Intervention Measure
CBT: cognitive behavioral therapy
IDI: In-Depth Interview
IMAGINE: Interactive Maternal Group for Information and Emotional Support
MB: Mothers and Babies Course
mHealth: mobile health
PHQ9: Patient Health Questionnaire
PSS-4: Perceived Stress Score
REDCap: Research Electronic Data Capture
SSB: Social Support Behavior

## Data Availability

The data sets analyzed during this study are available on GitHub [54].
