# Supplementary figures and images for: Acceptability and Utility of a Digital Group Intervention to Prevent Perinatal Depression in Youths via Interactive Maternal Group for Information and Emotional Support (IMAGINE): Pilot Cohort Study
Source: JMIR Form Res. 2024 Feb 2;8:e51066. doi: 10.2196/51066 (PMC10873795; doi:10.2196/51066)

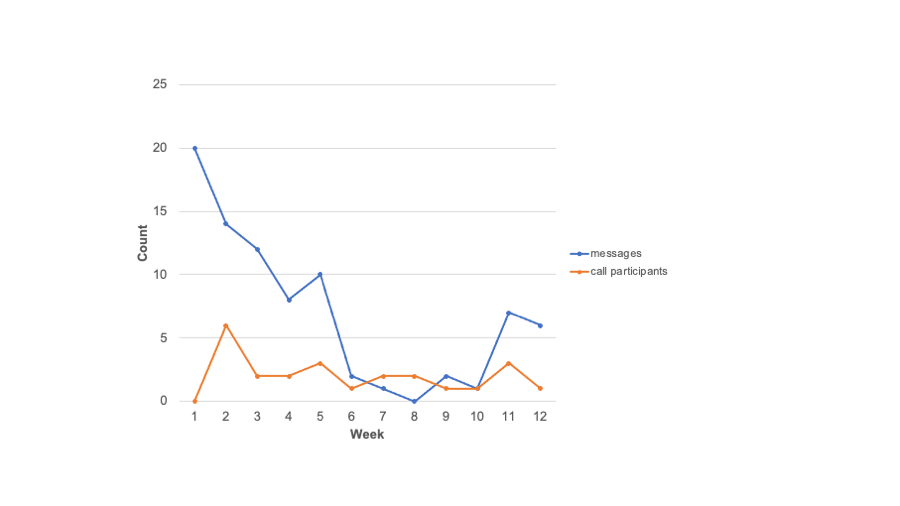

Supplement: Multimedia Appendix 1 [file formative_v8i1e51066_app1.png]

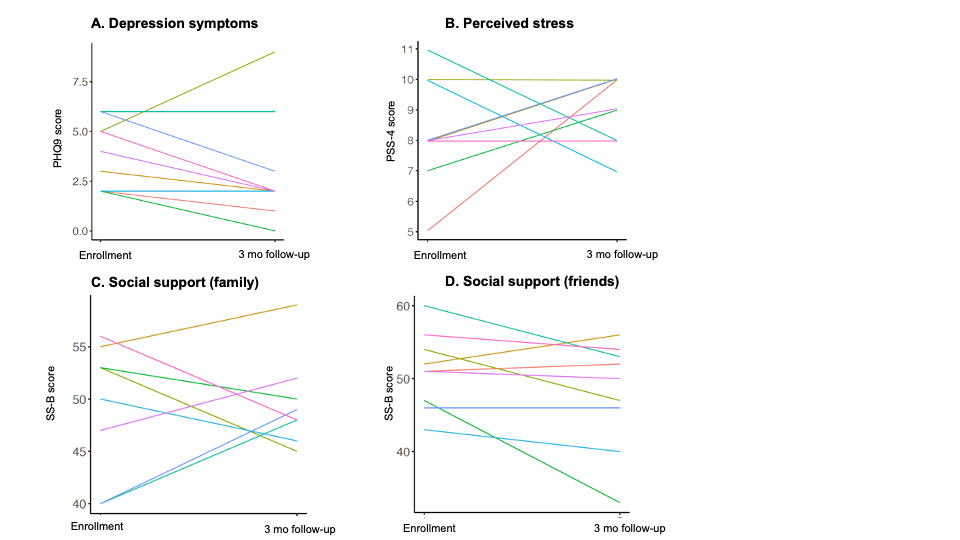

Supplement: Multimedia Appendix 2 [file formative_v8i1e51066_app2.png]
